# Association between ELMO1 gene polymorphisms and diabetic nephropathy in an Iranian population

**DOI:** 10.1186/s40200-016-0265-3

**Published:** 2016-10-07

**Authors:** Mohsen Mehrabzadeh, Parvin Pasalar, Mostafa Karimi, Maryam Abdollahi, Maryam Daneshpour, Effat Asadolahpour, Farideh Razi

**Affiliations:** 1 Department of Medical Biochemistry, Tehran University of Medical Sciences, Tehran, Iran; 2 Endocrinology and Metabolism Research Center, Endocrinology and Metabolism Clinical Science Institute, Tehran University of Medical Sciences, Tehran, Iran; 3 Diabetes Research Center, Endocrinology and Metabolism Clinical Sciences Institute, Tehran University of Medical Sciences, Tehran, 1411413137 Iran; 4 Endocrinology and Metabolism Clinical Sciences Institute, Shahid beheshti University of Medical Sciences, Tehran, Iran

**Keywords:** Diabetic nephropathy, Single nucleotide polymorphisms, ELMO1

## Abstract

**Background:**

Diabetic nephropathy (DN) is one of the leading causes of death in patients with type 2 diabetes mellitus (T2DM). Several genome-wide association studies have introduced ﻿Engulfment and Cell Motility 1 (ELMO1) ﻿as a candidate gene which is associated with DN. This study assessed the association of ELMO1 gene polymorphisms with DN in order to investigate the effects of ELMO1 gene on susceptibility to DN in an Iranian population.

**Methods:**

In the present study, 100 patients with T2DM, 100 patients with DN and 100 healthy subjects who were matched for sex were selected. Allele and genotype frequencies were determined by Tetra-ARMS PCR technique. In all groups, levels of FBS, creatinine, urea, HbA1C, urine levels of albumin creatinine ratio and glomerular filtration rate were measured.

**Results:**

A statistically significant association was shown between G allele of rs741301 (odds ratio (OR) = 1.7 [95 % CI 1.17–2.63]; *p* value = 0.005), and GG genotypes of rs741301 (OR = 2.5 [95 % CI 1.2–5.4]; *p* value = 0.01) and DN. A significant association was not detected between allelic and genotypic frequencies of rs1345365 and DN. Linkage Disequilibrium (LD) between two variants was weak (D' = 0.11, r2 = 0.008). rs1345365A/rs741301A haplotypes were more frequent in patients with T2DM as compared to DN (OR = 0.5 [95 % CI 0.3–0.7]; *p* value = 0.0006). Also, genotypes of variant rs741301 in all subjects had significant difference with respect to the mean of ACR (*p* Value < 0.05).

**Conclusion:**

This study first investigated the association of ELMO1 gene polymorphisms (rs741301) with DN in an Iranian population, supporting its key role as a candidate gene in the susceptibility to DN.

## Background

Type 2 diabetes mellitus (T2DM) has become a silent pandemic over the past few decades, resulting in remarkable loss in government’s economy, and has endangered human health [1, 2]. Patients with diabetes mellitus all over the world were estimated to be 173 million cases in 2002 and are anticipated to increase to 350 million cases by 2030 [3]. Microvascular complications of diabetes such as Diabetic Nephropathy (DN) can play a significant role in mortality of diabetes. DN as a progressive kidney disease affects almost 35 % of the diabetic patients irrespective of glycemic control [4]. It is the leading cause of kidney failure and end-stage renal disease worldwide [5] and also the most common cause of kidney transplant in Iran [6]. Further, patients with DN have an increased risk of cardiovascular mortality [7]. It is estimated that annual costs of hemodialysis treatment for each patient are an average of 11000 dollars in Iran [8, 9]. DN has been defined as increased protein excretion in urine and in lack of any kind of kidney disease [10].

Nowadays, it is generally accepted that DN is a multifactorial progressive disorder caused by the cooperation between environmental factors and genetic factors. The most important risk factors such as hyperglycemia, hypertension, diabetic duration, hyperlipidemia, chronic inflammation, familial clustering and genetic determinants can make a diabetic patient susceptible to DN. However, no clear etiology and pathogenesis have been found [11–13]. Outbreak of DN between ethnic groups and familial clustering affirms the presence of a genetic component [14–17]. Main part of the research on DN evaluates the genetic role in DN pathogenesis because elucidation of genes associated with DN can help for better understanding of disease molecular mechanism, complications, prevention and treatment [12]. As a result, early identification and more severe therapies can reduce the outcome of DN.

Genome-Wide Association Studies (GWAS) have reported some important genes all around the globe for resistance or susceptibility to DN [18]. ELMO1 (Engulfment and Cell Motility 1) as a new and strong candidate gene, located on chromosome 7p14.2-14.1, is used for cell motility and phagocytosis of apoptotic cells [19]. In spite of this, the precise role of ELMO1 in the development and progression of nephropathy attributed to T2DM is still unknown.

The first GWAS was conducted in Japanese individuals in 2005; over 80,000 SNP loci were evaluated and one SNP locus in the 18th intron of the ELMO1 gene was found to be strongly associated with DN [20]. Later studies have demonstrated that variants in the ELMO1 gene are associated with kidney disease attributed to T2DM in American Indians [21], European Americans [22] and Chinese population [23]. Also, recently large African American cohorts with type 2 diabetes have suggested that SNPs locus in the 13th intron of the ELMO1 gene was detected to be associated with DN [24]. Complications of T2DM are demonstrated to be more common among Asians compared to Western people [25] and this study was performed for the first time in Iran. The aim of the present study was to evaluate the possible association between the variants rs741301, rs1345365 and DN in a sample of the Iranian population.

## Methods

### Study subjects

This case–control study was performed between January and December 2015 in the Diabetes and Metabolic research Center affiliated to Endocrinology and Metabolism Research Institute (EMRI) of Tehran University of Medical Sciences (TUMS), Tehran, Iran. A total of 200 patients with T2DM were enrolled in the study that included 100 patients with nephropathy (DN) and 100 patients without nephropathy (DM). A control group consisting of 100 healthy controls (HC) was also included. T2DM was diagnosed by the criteria of the American Diabetes Association (ADA) [26]. Subjects with urinary albumin/creatinine ratio (ACR) ≥30 mg/g and protein excretion history caused by diabetes were considered as the DN group. DM group was indicated by ACR < 30 mg/g and ADA criteria for diabetes. Subjects of the control group were healthy population without diabetes and any kind of kidney disease. Exclusion criteria in the present study were: subjects of non-Iranian origin, patients with Type 1diabetes, diabetes duration less than 5 years, HbA1C > 9 %, urinary tract infection, uncontrolled hypertension, pregnancy, hematuria, smoking and any other cause of protein excretion except diabetes type 2. Ages of subjects in all the population were between 35 and 75. The groups were matched for sex. The study was approved by the Ethics Committee of EMRI. After describing the aims of the study, a written informed consent was acquired from each subject.

### Clinical characteristics of subjects

Several demographic and anthropometric measurements were taken. Blood pressure was measured after 20 min of rest. The weight and the height were measured and the body mass index (BMI) was calculated (kg/m^2^). Urine and fasting blood samples were obtained from the subjects for biochemical analysis and DNA extraction. Glycosylated hemoglobin (HbA1C) (TOSOH G8, Tokyo, Japan) and Fasting Blood Sugar, urea, creatinine, and also urine albumin creatinine ratio (Pars Azmun, Tehran, Iran) were measured. Estimated glomerular filtration rate (eGFR) was calculated using Cockroft and Gault equation [27].

### Genotyping

We selected the variants rs741301 and rs1345365 of ELMO1 gene, which were presented as common SNPs by some considerable genome-wide association studies (GWAS) in other populations. Genomic DNA was extracted from peripheral blood lymphocytes in the buffy coat using Bio Basic DNA isolation kit (Bio Basic Inc., Markham, Canada) in accordance with manufacturer’s instructions. The quantity and quality of DNA were examined with spectrophotometer and electrophoresis. SNPs genotyping was carried out by tetra-ARMS-PCR, which is a rapid, reliable, and cost-effective method for the detection of SNPs [28, 29]. Outer and inner primers are shown in Table 1. PCR product sizes for the variant rs741301 polymorphism were: 407 bp for two outer primers (control bands), 187 bp for G allele and 273 bp for A allele. Also, product sizes in order to check the variant rs1345365 polymorphism were: 692 bp for two outer primers (control bands), 284 bp for G allele and 461 bp for A allele. PCR for ELMO1 amplification was carried out in 0.2 ml tubes which totally contain 25 μL reaction mixture. The reaction mixture was made by adding 2 μL template DNA, 1 μL for inner primers and 0.5 μL for outer primers (10 μM), 12.5 μL available PCR premix (Taq DNA Polymerase Master Mix Red, Ampliqon, Skovlund, Denmark) according to the manufacturer recommended protocol and 7.5 μL distilled water. The PCR cycling condition for the detection of the variant rs741301 polymorphism was 95 °C for 5 min followed by 35 cycles, including denaturation at 94 °C for 35 s, annealing at 63.5 °C for 30 s, and extension at 72 °C for 40s. The PCR condition for the detection of the variant rs1345365 polymorphism was 5 min at 95 °C followed by 35 cycles of 35 s at 95 °C, 30 s at 66 °C and 30 s at 72 °C with a final extension at 72 °C for 5 min in order to complete the extension of all PCR fragments. The PCR products were detected by electrophoresis on a 1.8 % agarose gel. Then, it was visualized by transillumination with UV light and finally photo was taken. Negative and positive controls were used in each genotyping run and 10 % of the randomly selected samples were re-genotyped in order to verify the initial results, with 100 % concordance. Also, genotypes were confirmed by randomly selecting some samples for DNA Sequencing.

**Table 1:** Primers used for polymorphism determination of ELMO1

Primers	rs741301	rs1345365
Forward inner	5′ATAGCAATAGATTTTATGAGGTGGTAGT 3′	5′GCCACTTCTTCCCCTACAACATTGA 3′
Reverse inner	5′TCATTAGTGATAACATAACCTCTGGTAG 3′	5′GCCAGTGAGAGAGTAATACTATTACGTTC3′
Forward outer	5′AGTCTTGAGGATGAATGAATTCTAGG 3′	5′TGCCATAGGTACTGCTTCTCTGAGT 3′
Reverse outer	5′TGTCCTAACAATTCGTTCATGATTAATG 3′	5′CTGAAGTCTAGTAAGAGCTCAAGGTCAGT 3′

### Statistical analysis

Statistical analyses were performed using the SPSS version 22.0 software for windows (SPSS Inc, Chicago, IL). Each SNP was evaluated for Hardy-Weinberg equilibrium (HWE) by using an HWE calculator [30]. Fisher exact test was used in order to compare genotypes and alleles between groups. Mann–Whitney test was used in order to compute the continuous variables. Odds Ratio (OR) and the 95 % confidence intervals (CI) were calculated by using Unconditional logistic regression in order to examine the association between genotypes and DN. Also, adjusting for potential confounders such as sex, age and diabetes duration was performed by Multivariate logistic regression. Clinical parameters of the subjects were evaluated as Mean ± SD. One-way ANOVA was used in order to calculate the significance of the difference among the clinical parameters of groups. HWE, haplotype construction and frequency analysis were evaluated with SHEsis software [31]. A *p* value < 0.05 was considered to be statistically significant.

## Results

The demographic and clinical characteristics of groups are shown in Table 2. Age, systolic blood pressure, body mass index (BMI), duration of diabetes, fasting blood sugar, HbA1C, serum creatinine, serum urea, and ACR were higher in DN group compared to DM and HC groups. Furthermore, eGFR was lower in DN subjects than DM & HC.

**Table 2:** Demographic and clinical characteristics of groups’ subjects

Parameters	DN (*n* = 100) mean ± SD	DM (*n* = 100) mean ± SD	HC (*n* = 100) mean ± SD	*p* value
Age (years)	61.5 ± 8.5	57.36 ± 8.1	50.5 ± 10.7	*A
Duration of diabetes (years)	12.01 ± 7.6	10.20 ± 6.9	-	‡
BMI (Kg/m^2^)	29.09 ± 5.3	28.7 ± 4.5	27. 94 ± 4.9	‡
SBP (mmHg)	125 ± 13.08	120.7 ± 20	115.2 ± 12.6	*A
DBP (mmHg)	77.2 ± 8.8	76.5 ± 7.9	79.3 ± 6.2	‡
FBS (mg/dl)	144.06 ± 54.3	140.6 ± 45	92.4 ± 10.9	*A
HbA1C (%)	7.2 ± 0.70	7.09 ± 0.73	5.5 ± 0.58	*A
creatinine (mg/dl)	1.2 ± 0.41	1.1 ± 0.27	1 ± 0.17	*A
Urea (mg/dl)	42.3 ± 17.3	36.7 ± 13.7	31.3 ± 10.1	*A
eGFR (L/min/1.73 m2)	65.9 ± 20.6	75.2 ± 20.7	78.8 ± 17.3	*B
ACR (μg/mg)	83.3 ± 64.6	23.7 ± 5.5	21.3 ± 7.7	*B

*DN* Type 2 diabetes with Nephropathy, *DM* Type 2 diabetic without Nephropathy, *HC* Healthy control, *BMI* Body Mass Index, *SBP* Systolic Blood Pressure, *DBP* Diastolic Blood Pressure, *eGFR* estimated Glomerular Filtration Rate, *FBS* Fasting Blood Sugar, *ACR* urine Albumin/Creatinine ratio

*means p value <0.05, ‡ means *p* value > 0.05, A = Significant difference between DM/DN and HC, B = Significant difference between DN and HC/DM

Serum urea adjusted by age and sex. FBS, HbA1C and serum creatinine adjusted by duration of diabetes

The allele and genotype frequencies of two variants in diabetic patients and healthy controls are shown in Tables 3 and 4. Two SNPs had minor allele frequency of more than 0.05 and were matched with HWE. GG genotypes and G allele of the variant rs741301 were more frequent in DN group as compared to DM. Moreover, G allele of the variant rs741301 was more frequent in DN as compared to HC. There was no significant difference in allele and genotypes frequencies of the variant rs1345365 between DN and DM or between DN and HC. Then, we applied two SNPs in an unconditional logistic regression analysis, after checking for age, sex, creatinine, blood pressure, BMI and so on.

**Table 3:** Allele and genotypes frequencies distribution of ELMO1 variants in DN and DM subjects

SNP ID	Allele/Genotype	DN (*n* = 100) N (%)	DM (*n* = 100) N (%)	*p* value	OR (95 % CI)
rs1345365	A	143 (71.5)	153 (76.5)	0.2	0.7 (0.4–1.2)
	G	57 (28.5)	47 (23.5)	0.2	1.2 (0.8–2)
	AA	51 (51)	58 (58)	0.3	0.7 (0.4–1.3)
	AG	41 (.41)	37 (37)	0.5	1.1 (0.6–2)
	GG	8 (8)	5 (5)	0.3	1.6 (0.5–5.2)
HWE		0.9	0.7		
rs741301	A	106 (53)	133 (66.5)	0.005	0.5 (0.3–0.8)
	G	94 (47)	67 (33.5)	0.005	1.7 (1.17–2.63)
	AA	32 (32)	45 (45)	0.05	0.5 (0.3–1)
	AG	42 (42)	43 (43)	0.8	0.9 (0.5–1.6)
	GG	26 (26)	12 (12)	0.01	2.5 (1.2–5.4)
HWE		0.1	0.7		

*HWE* Hardy-Weinberg equilibrium, *DN* Type 2 diabetes with Nephropathy, *DM* Type 2 diabetes without Nephropathy

**Table 4:** Allele and genotypes frequencies distribution of ELMO1 variants in DN and HC subjects

SNP ID	Allele/Genotype	DN (*n* = 100) N (%)	HC (*n* = 100) N (%)	*p* value	OR (95 % CI)
1345365	A	143 (71.5)	138 (69)	0.2	1.1 (0.7–1.7)
	G	57 (28.5)	62 (31)	0.2	0.8 (0.5–1.3)
	AA	51 (51)	47 (47)	0.3	1.1 (0.6–2)
	AG	41 (41)	44 (44)	0.1	0.8 (0.5–1.5)
	GG	8 (8)	9 (9)	0.7	0.8 (0.3–2.3)
HWE		0.9	0.7		
rs741301	A	106 (53)	129 (64.5)	0.01	0.6 (0.4–0.9)
	G	94 (47)	71 (35.5)	0.01	1.6 (1.1–2.5)
	AA	32 (32)	44 (44)	0.08	0.5 (0.3–1)
	AG	42 (42)	41 (41)	0.8	1 (0.5–1.8)
	GG	26 (26)	15 (15)	0.05	2 (1–4.1)
HWE		0.1	0.2		

*HWE* Hardy-Weinberg equilibrium, *DN* Type 2 diabetes with Nephropathy, *HC* Healthy Control

Linkage Disequilibrium (LD) between two ELMO1 SNPs, rs741301 and rs1345365 was tested by using SHEsis software and it was estimated that LD between two ELMO1 SNPs was weak (D' = 0.11, r2 = 0.008). Also, haplotypes frequencies were tested between DN and DM subjects and the results are shown in Table 5. rs1345365A/rs741301A haplotypes were more frequent in DM as compared to DN and they were found to be protective against DN. rs1345365A/rs741301G haplotypes were more frequent in DN as compared to DM.

**Table 5:** Haplotypes frequencies of ELMO1 gene polymorphisms between DN and DM subjects

Haplotypes	DN (*n* = 100) N (%)	DM (*n* = 100) N (%)	Chi2	*p* value	OR (95 % CI)
rs1345365A/rs741301A	77 (38.5)	111 (55.5)	11.6	0.0006	0.5 (0.3–0.7)
rs1345365A/rs741301G	66 (33)	42 (21)	7.3	0.006	1.85 (1.1–2.9)
rs1345365G/rs741301A	29 (14.5)	22 (11)	1.1	0.2	1.3 (0.7–2.4)
rs1345365G/rs741301G	28 (14)	25 (12.5)	0.1	0.6	1.14 (0.6–2.03)

*DN* Type 2 diabetes with Nephropathy, *DM* Type 2 diabetes without Nephropathy

Finally, we evaluated the association of several clinical parameters in all subjects with respect to the variants genotypes. Results showed that each genotype of the variant rs1345365 in all subjects was not associated with various clinical parameters. But after using the post-hoc bonferroni test, we found that GG genotype vs AA or AG genotypes of the variant rs741301 in all subjects had significantly higher level of ACR (*p* value = 0.03).

## Discussion

In the present study, we investigated ELMO1as a candidate gene for susceptibility to DN in Iranian individuals; a population with a high incidence of DN. Our analysis suggested an association between allelic and genotypic frequencies of the variant rs741301 and susceptibility to DN. In fact, we detected that G allele (OR = 1.7 [95 % CI 1.17–2.63], *p* = 0.005) and GG genotype (OR = 2.5 [95 % CI 1.2–5.4] *p* = 0.01) might predispose an Iranian patient with T2DM to DN. Over the last decade, GWAS and replication studies have produced impressive results upon the genetic basis of DN. GWAS in Japanese type 2 diabetic patients (546 DN cases and 334 type 2 diabetic controls) [20], replication studies in GoKinD collection study (558 DN cases and 820 type 2 diabetes controls) [22] and American Indian study (107 cases with diabetic ESRD and 108 control subjects with T2DM) [21], African American cohorts (1160 controls and 1136 cases include End stage renal disease and T2DM) [24], Chinese population (123 DN cases and 77 type 2 diabetic controls) [23] and Indian population (202 cases with DN and 215 controls with T2DM) [32] support the key role of ELMO1 as a susceptible gene in DN. Although most studies identified ELMO1 as a candidate gene for DN, results showed that risk SNPs and risk allele are not similar in all populations [33].

Most studies represented rs741301 polymorphism as the strongest SNP associated with DN. In 2005, GWAS was first done in Japanese T2DM patients and the obtained results identified the strongest association between rs741301polymorphism and DN. They showed that G allele in rs741301 and GG genotype are associated with risk of DN in Japanese patients, which is consistent with our results [20]. Risk allele in rs741301 in Chinese population was opposite to that identified in our study as the A allele in rs741301 was associated with risk of DN in Chinese people whereas in Iranian population, it was a protective allele for T2DM patients against progressing to DN. Moreover, a significant difference was found between DN and DM with respect to allelic and genotypic frequencies of rs741301 and rs10951509 polymorphism [23]. American-Indian and Mexican-American population studies failed to show the significant association between allelic and genotypic frequencies of the variant rs741301 and susceptibility to DN [21, 34]. A meta-analysis of genetic associations in diabetic nephropathy showed that rs741301 of ELMO1 was associated with diabetic nephropathy in Asians with type 2 diabetic nephropathy (OR 1.58 [95 % CI 1.28–1.94]) [35]. Indian population study showed that GG genotype frequencies were higher in diabetic versus HC groups (*p* value < 0.05), which is consistent with our results [32]. The marker rs741301 located in intron 18 of ELMO1 gene was in weak Linkage Disequilibrium (LD) with marker rs1345365 located in intron 13 of ELMO1 gene (D' = 0.11, r2 = 0.008), which is consistent with the result of the Chinese study as two SNPs had weak LD together (D' = 0.3).

Also, in the present study, we did not find any significant difference between case and control groups with respect to allelic and genotypic frequencies of the variant rs1345365 of intron 13, which is consistent with the Chinese study [23], Mexican study [36] and opposite to American Indians study as the A allele in rs1345365 was associated with the increased risk of DN in American Indian people. African American cohorts showed that four SNPs located in intron 13 (rs1345365, rs1981740, rs10951509 and rs2058730) are associated with T2DM-ESRD [24]. Haplotypes analysis suggested rs1345365A/rs741301A as a protective haplotype (OR = 0.5 [95 % CI 0.3–0.7] *p* value = 0.0006) for Iranian patients with T2DM against progressing to DN, which is inconsistent with the Chinese study as this haplotype has been identified as a risk haplotype for DN [23]. Also, our results presented rs1345365A/rs741301G as a risk haplotype (OR = 1.85 [95 % CI 1.1–2.9], *p* value = 0.006) for Iranian patients with T2DM progressing to DN. These results showed that rs741301 polymorphism may play an important role in increasing the risk of DN. Results of studies maybe conflicting and the reason may be related to unknown etiology of DN, multiple genetic factors, environmental and racial differences and inappropriate sample sizes.

In 2006, Shimazaki and colleagues found that expression of ELMO1 gene increased in high glucose conditions. Subsequently, it terminated to increased expression of extracellular matrix genes and decreased expression of metalloproteinase gene and cell adhesive properties might make diabetes progress to DN. The exact mechanism of ELMO1 role in expression of these genes is still unknown, but last studies suggested that ELMO1 cooperates with Dock180 and they activate Rac-1 and this complex leads to increased expression of extracellular matrix gene. Shimazaki showed that there are not significant effects of these factors on the expression of extracellular matrix gene [19, 37].

The present study evaluated for the first time the genetic variants of ELMO1 in an Iranian subjects with or without T2DM. The limitations of this study were the relatively small sample size, especially in DN group and also lack of diabetic patients with ESRD.

## Conclusions

ELMO1 may play a role in diabetic kidney disease in Iranian individuals as a population with high prevalence of T2DM, DN and ESRD. We found that G allele and GG genotypes of the marker rs741301 may predispose a diabetic patient to progressing to DN. Also, the association between the marker rs741301 and mean of ACR implicates this gene in susceptibility to DN. As a result, ELMO1 could be selected as an appropriate target in order to prevent and treat DN.

## Abbreviations

BMI: Body Mass Index
DBP: Diastolic Blood Pressure
DM: Type 2 diabetic without Nephropathy
DN: Type 2 diabetes with Nephropathy
eGFR: Estimated Glomerular Filtration Rate
EMRI: Endocrinology and Metabolism Research Institute
ESRD: End Stage Renal Disease
FBS: Fasting Blood Sugar
HC: Healthy control
HWE: Hardy-Weinberg equilibrium
SBP: Systolic Blood Pressure
TUMS: Tehran University of Medical Sciences
